# Cellulose Acetate Membranes from Sisal Fiber Applied for Furfural Recovery

**DOI:** 10.1021/acsomega.6c00887

**Published:** 2026-04-29

**Authors:** Franklin Damião Xavier, Maria Gardennia Fonseca, Alessandro Silva Guedes Lima Bruno, Sandro Marden Torres, Marta Maria Conceição

**Affiliations:** † PPGQ/CCEN, Universidade Federal da Paraíba, 58051-970 João Pessoa, PB, Brazil; ‡ Departamento de Química/CCEN, Universidade Federal da Paraíba, 58051-970 João Pessoa, PB, Brazil; § Departamento de Engenharia Mecânica/CT, Universidade Federal da Paraíba, 58051-900 João Pessoa, PB, Brazil; ∥ Departamento de Tecnologia de Alimentos/CTDR, Universidade Federal da Paraíba, Av. dos Escoteiros, sn. Mangabeira VII, 58058-600 João Pessoa, PB, Brazil

## Abstract

Membrane separation is an effective method for recovering biomass in biorefineries. Cellulose acetate (CA) membranes exhibit excellent separation capabilities and high selectivity. The use of lignocellulosic materials, such as sisal fiber, for the production of CA membranes represents a unique opportunity in this context. This study aimed to develop sisal fiber-derived CA membranes under optimized synthesis conditions for the efficient removal of furfural from lignocellulosic hydrolysates. The biomass was subjected to chemical treatments (organosolv and dilute acid) to isolate the cellulose. An experimental design approach was used to determine the optimal conditions for cellulose recovery, yielding a product containing 2.4% residual impurities. This cellulose was subsequently acetylated to produce CA, a conversion confirmed by spectroscopic analysis. CA and CA/PEG-400 membranes (incorporating poly­(ethylene glycol) 400 into their composition) were synthesized using the phase inversion method. All membranes exhibited a porous, asymmetric structure. Membranes containing PEG-400 within the polymer matrix displayed a higher porosity percentage, lower permeate flux, and higher furfural recovery. The inclusion of PEG-400 resulted in a membrane with a smoother surface and smaller pores—findings corroborated by scanning electron microscopy data. The experimental condition involving the lowest synthesis temperature (5 °C) and the presence of PEG-400 yielded the highest furfural retention, reaching 92%.

## Graphical abstract

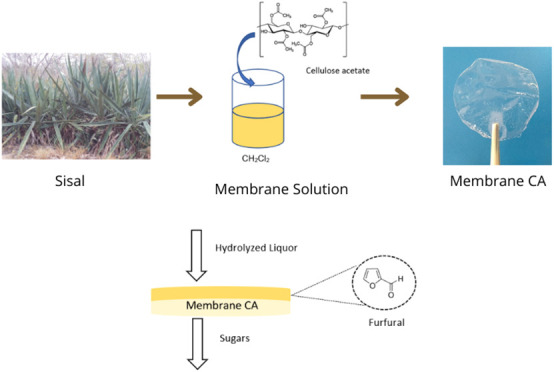

## 1 Introduction

Current research aims to find new sources of renewable energy as well as sustainable and affordable solutions for transportation and infrastructure systems that would benefit society.^1^ Most human activities, such as agriculture, transportation, and industrialization, generate significant environmental impacts, emitting contaminants into the environment. Biomass can come from various sources, including agricultural products, residual biomass, livestock byproducts, and urban waste, so it an excellent renewable hydrocarbon resource. Among these biomass sources, lignocellulosic materials stand out. These compounds are some of nature’s most abundant renewable organic resources.^2,3^

Sisal is considered a high-value lignocellulosic material due to the amount of carbohydrates in its structure: It is roughly 70% cellulose and hemicellulose. Approximately 4.5 million tons of sisal fiber are extracted annually from the leaves of the sisal plant (*Agave sisalana*), grown in tropical countries. A typical sisal plant contains 200–250 sisal leaves, 4% of which are used for fiber production.^4^ Sisal biomass is considered a renewable raw material that can be transformed into various chemical products, membranes, and biofuels. In biorefineries, biomass is converted using various techniques, such as biochemical and thermochemical processes, which are used to synthesize biochemical products.^5,6^

Lignocellulosic biomass is composed of cellulose, hemicellulose, and lignin. These biocomponents have been used to produce bioethanol, biochar, biohydrogen, and biobutanol, among other products.^7^ For biofuel production, cellulose and hemicellulose require pretreatment for depolymerization. Among the pretreatments, acid hydrolysis targets either cellulose or hemicellulose, depending on the experimental conditions. While dilute acid targets xylose, alkaline treatment primarily targets lignin, exposing carbohydrates. Although hydrolysis is important for depolymerization, it can lead to the release of compounds that inhibit the bioconversion process. These products are generated from the decomposition of sugars present in hemicellulose and lignin. The main inhibitors are acetic acid, 5-hydroxymethylfurfural (HMF), and furfural. Cellulose is generally degraded into glucose, which is isomerized into fructose, which is then hydrolyzed into HMF. Meanwhile, hemicellulose is converted to furfural.^8,9^

Furfural is classified as a furan and is formed by the decomposition of pentoses in processes involving high temperatures and pressures. While it acts as an inhibitor of fermentation processes in biorefineries, once isolated, it can be applied to a wide range of chemical products. Its structure includes a conjugated double bond based on an aldehyde, which can undergo various reactions to produce biofuels and additives, and its derivatives are used in the production of resins, plastics, pharmaceuticals, and pesticides. The major obstacles to furfural production lie in the low yield and selectivity of the reaction, in addition to the environmental problems arising from its synthesis and purification, such as the use of homogeneous acid catalysts, which produces large amounts of wastewater.^10,11^

Furfural is industrially recovered through conventional separation technology, that is, fractional distillation. This process is often costly and has a low yield.^12^ Heterogeneous catalysts have been widely used, but this approach leads to the formation of byproducts that interfere with separation and pose a significant barrier to commercial scalability. Technologies such as vacuum distillation, solvent extraction, or membrane separation have been employed to separate the bioproduct. The use of membranes could allow for furfural recovery at a lower operating cost and with the use of a smaller system.^13,14^

Therefore, there are high demand for a simpler, more energy-efficient and environmentally friendly pathway in separation. Membrane separation has proven to be highly effective in biomass processing in biorefineries to remove inhibitors and to recover products. It acts as a preliminary purification step, ensuring that desirable components are retained while undesirable inhibitors are eliminated.^15^ Cellulose acetate (CA) membranes have demonstrated high stability and selectivity with good sugar recovery. According to prior research, nanofiltration processes can remove over 99% of organic acids and furans from lignocellulosic hydrolysates, so they represent a viable detoxification method for biorefinery applications.^13,16^

This study aimed to develop a CA membrane from sisal fiber to evaluate its potential application in removing furfural from biomass-derived liquors, which are part of the biotechnological alcohol production process and serve as an intermediate purification step. This is considered one of the major bottlenecks faced in biorefineries, as furfural drastically reduces the conversion of carbohydrates into commercially valuable materials. In addition to its role in this stage, the recovery of furfural is also highly advantageous, as it is a byproduct utilized in other industrial processes. Specifically, the optimal synthesis conditions, including the synthesis temperature were examined. Moreover, poly­(ethylene glycol) (PEG) 400 was added to the membrane to evaluate its effects on the structure, pore morphology, and furfural separation performance of the membrane.

## 2 Materials and Methods

### 2.1 Materials

Sisal fiber was acquired during sisal leaf defibration for fiber production on a farm located in the state of Paraíba, Brazil. The following reagents were used: sulfuric acid (Neon, >95%), sodium hydroxide (Dinâmica, 97%), glacial acetic acid (Vetec, 99.7%), acetic anhydride (Synth, 97%), dichloromethane (Synth, 99.5%), sodium chlorite (Sigma-Aldrich, >80%), ethyl alcohol (Synth, 99.5%), PEG 400 (Synth, 99.5%), and nitric acid (Vetec, 65%).

### 2.2 Delignification and Experimental Design

The sisal fiber was dried in a Solab SL-102 air circulation and renewal oven at 60 °C for 8 h. Then, the material was ground in a Solab SL-31 knife mill for subsequent particle size distribution. To begin cellulose isolation/recovery, the biomass underwent an organosolv delignification process, where 4.0 g of the sisal sample *in natura* was weighed and 80 mL of an ethanol/nitric acid solution (80/20, v/v) was added. The mixture was placed in a reflux system and heated for 3 h. Finally, the biomass was filtered and washed with distilled water.^17^

To finalize the isolation of cellulose present in the sisal biomass, it was pretreated with dilute sulfuric acid to partially or completely remove the hemicellulosic fraction. For this purpose, a 2^3^ factorial design with three replicates at the central point was employed. The effects of the input variables—the sulfuric acid concentration (1%, 2%, and 3%, v/v), temperature (100, 110, and 120 °C), and time (30, 45, and 60 min)—on the solubilization of xylose present in the biomass were analyzed. The experiments were carried out in a pressurized stainless-steel reactor (Biofoco) lined with Teflon with an operating volume of 700 mL and 1:10 (w/v) ratio of sisal to acid solution. The response variables were the concentrations of xylose and glucose obtained in the hydrolyzed liquors, which were quantified through high-performance liquid chromatography (HPLC).^9^

### 2.3 Determination of the Kappa Number and the Lignocellulosic Composition

To determine the effectiveness of cellulose recovery, TAPPI T19 M-54, TAPPI 203 cm-99, and TAPPI 222 were used to quantify the holocellulose; α-, β-, and γ-cellulose; and lignin contents, respectively, before and after chemical pretreatments. In addition, the Kappa number was used to evaluate the effectiveness of the treatment. It was determined based on the Brazilian standard ABNT ISO 302:2005 for cellulose pulps.^18,19^

### 2.4 Preparation of CA Membranes

After the cellulose was isolated, it underwent a homogeneous acetylation process to produce cellulose triacetate. Two grams of cellulose were weighed; then, 40 mL of glacial acetic acid was added, and the solution was stirred for 30 min at room temperature. Subsequently, 0.3 mL of sulfuric acid and 19 mL of acetic acid were added. The mixture was stirred for 25 min, followed by the addition of 20 mL of acetic anhydride, followed by stirring for another 30 min. After this period, the reaction was stopped, and the solution was incubated for 24 h. Then, distilled water was added until a precipitate formed, and the sample was filtered and washed until its pH was close to that of water. The retained CA was oven-dried at 60 °C and characterized by Fourier-transform infrared (FTIR) spectroscopy.^20^

The phase inversion method was used to produce the CA membranes. The membranes differed in terms of the temperature of the coagulation bath and the presence or absence of PEG 400 as an additive. Briefly, 3 g of CA from sisal fiber was weighed and added to 50 mL of dichloromethane. The mixture was stirred for 6 h, then placed in an ultrasonic bath for 10 min to remove air bubbles. The solution was spread on a glass plate. After waiting 5 min for the solvent to evaporate, the plate was immersed in a coagulation bath containing deionized water for 2 h to complete the phase inversion process.^21,22^

### 2.5 Membrane Filtration Experiments

#### 2.5.1 Porosity

The membranes were cut into 4 cm^2^ squares and stored in distilled water. Then, they were superficially dried using absorbent cloth and weighed to determine the wet mass (*W*_0_). The process was repeated for all samples. Subsequently, the wet membrane was placed in an air circulation oven at 80 °C for 24 h until it reached a constant mass.^23^ The dewetted membrane was weighed and the porosity calculated using (eq 1)­

1 $$P\left(\%\right)=\left(\frac{W_{0}-W_{1}}{A\times h}\right)\times 1000$$

where *P* is membrane porosity, *W*_0_ is the wet membrane weight (g), *W*_1_ is the dry membrane weight (g), *A* is the membrane surface area (cm^2^), and *h* is the membrane thickness (mm).

#### 2.5.2 Permeate Water Vapor Flux

The permeate water vapor flux was determined using a Payne cup and following the ASTM E96 standard.^24^ Fifteen milliliters of distilled water was added to a Payne cup. The membranes were cut into discs with a diameter equal to that of the container and inserted, was sandwiched between two sealing rings. The assembly was weighed on an analytical balance and placed in a desiccator. This pressure gradient generated within the desiccator allows water vapor to permeate the polymer film. The mass variation of the system was monitored over a 1 h interval between each weighing. A graph of the mass variation as a function of time was constructed from the data obtained. The equation of the line was determined using linear regression, and the slope was used to calculate the water vapor flux using (eq 2)­

2 $$J=\frac{\Delta m}{\Delta t}.\frac{1}{A}$$

where *J* is the vapor flux (mg h^–1^ cm^–2^), Δ*m* is the mass variation (mg), Δ*t* is the time variation (h), and *A* is the membrane area (cm^2^).

#### 2.5.3 Filtration Studies

A dead-end filtration system was used to examine furfural recovery from lignocellulosic hydrolysates. All experiments were conducted at a fixed pressure of 1 bar and room temperature. The hydrolyzed liquor used in the tests was obtained from the hydrolysis of raw sisal fiber using 4% sulfuric acid at a temperature of 150 °C. After hydrolysis, the composition was determined using HPLC. Prior to conducting the performance tests, the pH of the liquors was adjusted to 5 using sodium hydroxide to prevent physical wear on the membrane caused by the acidic environment of the initial liquor, which had a pH of 0.5. Membrane performance was assessed by measuring the furfural recovery rate at the end of the tests, where the percentage removal of the CA membrane was calculated based on the difference between the initial hydrolysate and the permeate solutions.

### 2.6 Characterization

#### 2.6.1 FTIR Spectroscopy

The chemical composition of cellulose and its derivatives were analyzed with FTIR spectroscopy in the range of 4000–400 cm^–1^ using KBr and a Shimadzu IR Prestige spectrophotometer.

#### 2.6.2 Scanning Electron Microscopy

A Quanta 450 scanning electron microscope (FEI) with an energy dispersive X-ray spectroscopy system (EDS) was used to evaluate the morphology and changes in the physical structure of the membranes. Prior to analysis, the membranes into small squares, which were freeze-dried and then coated with gold. The analysis was performed at a voltage of 1 kV.

#### 2.6.3 Atomic Force Microscopy

The surface morphology of the membranes was analyzed using a scanning probe microscope (Shimadzu model SPM-9600). The membrane was cut into a 1 cm^2^ square, which was inserted into the sample holder. The scanned surface was analyzed in 1 μm × 1 μm images.

#### 2.6.4 HPLC

A 1260 Infinity liquid chromatograph (Agilent Technologies) equipped with an autosampler (model G1329B) coupled to a diode array detector (DAD) (model G1315D) and a refractive index detector (RID) (model G1362A) was used for HPLC. After filtering the same through a 0.22-μm nylon membrane, 10 μL was injected into the system. The ion exchange column used was an Agilent Hi-Plex H (300 mm × 7.7 mm) with an 8.0 μm particle size and a PL Hi-Plex H guard column (5 mm × 3 mm). The column compartment temperature was maintained at 70 °C and the RID flow cell was maintained at 50 °C. The mobile phase was 4.0 mmol L^–1^ solution of H_2_SO_4_ in ultrapure water, applied at a flow rate was 0.5 mL min^–1^.

## 3 Results and Discussion

### 3.1 Cellulose Isolation

The cellulose from the sisal fiber underwent organosolv treatment to remove the lignin fraction. Each pretreatment has advantages and disadvantages. For example, alkaline treatment is effective for solubilizing lignin, but it generates residues that inhibit biofermentation. Organosolv treatment provides selective lignin extraction—in some cases, the extracted lignin can reach 94% purity.^25^ After organosolv treatment, the biomass was pretreated with dilute acid to remove the hemicellulosic fraction.^26^ Hemicellulose is a heterogeneous and branched polymer composed primarily of xylose, with smaller amounts of glucose and arabinose. Thus, the 2^3^ factorial design evaluated the influence of acid treatment on xylose solubilization and, consequently, the removal of the hemicellulose fraction from the structure. The results and experimental conditions are shown in Table 1.

**Table 1:** Factorial Design and Sugar Concentrations for Sisal Fiber

experiment	acid concentration (%)	temperature (°C)	time (min)	xylose (g L^–1^)
1	1	100	30	0.68
2	3	100	30	13.11
3	1	120	30	13.26
4	3	120	30	14.58
5	1	100	60	0.97
6	3	100	60	7.68
7	1	120	60	6.45
8	3	120	60	14.79
9	2	110	45	12.09
10	2	110	45	11.94
11	2	110	45	12.75

Condition 8 of the 2^3^ factorial design led to the highest xylose removal concentration (14.79 g L^–1^) in the hydrolyzed liquor. Previous studies reported a xylose removal concentration of 12.4 g L^–1^ when using sisal bagasse as a carbohydrate source and 1.1 g L^–1^ at a temperature similar to that used in the present work (120 °C) and a longer reaction time (80 min).^9,27^ In another study that aimed to optimize acid hydrolysis of sisal fiber, Medeiros et al.^27^ obtained a higher xylose removal concentration of 16 g L^–1^, but they used a temperature above 150 °C and a higher sulfuric acid concentration. The present work obtained a very similar xylose concentration using a milder temperature (120 °C) and a lower sulfuric acid concentration (3%).

A Pareto chart is widely used to represent the significance of parameters and their interactions in an experimental design. In Figure 1a, the horizontal bars of the Pareto chart display the factors for the proposed experimental design in decreasing order of importance. Any parameter that crosses the dashed line is considered significant according to the adopted confidence level (95% for this study).^28^ The concentration of xylose removed from sisal fiber was affected by all independent variables. Increasing the acid concentration and temperature increased the concentration of solubilized xylose. It is also important to highlight the statistically significant interaction between the three parameters. Of the second-order interactions, only temperature and acid concentration were significant.

**Figure 1:**
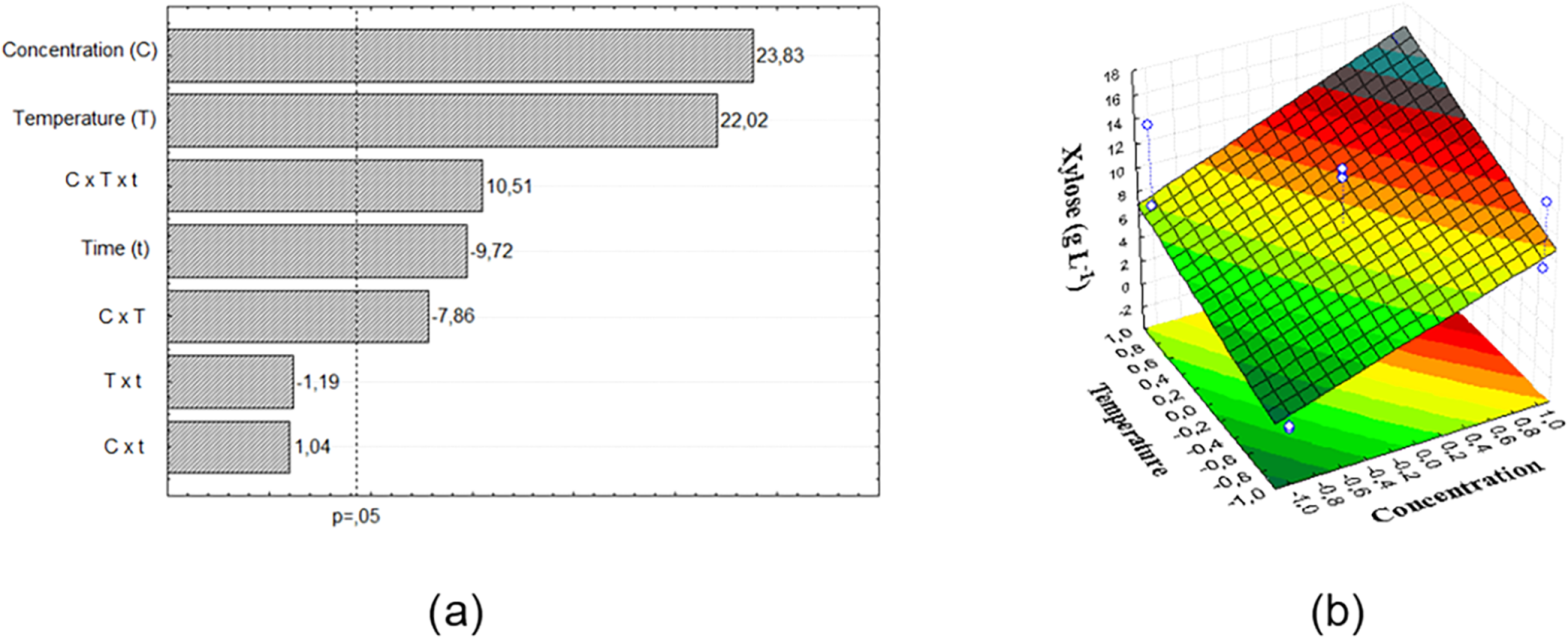
Pareto chart (a) and response surface of sisal fiber (b).

Based on the significance of the model, the equation that reproduces the xylose concentration predicted by the model as a function of the coded variables was determined: Xylose = 9.85 + 3.60 C + 3.33 T – 1.46 t – 1.18 TC + 1.59 CTt. An analysis of variance (ANOVA) was performed to assess the response to the xylose concentration removed by acid treatment of the sisal fiber, considering only the parameters that were significant. For this ANOVA, the calculated F value was 1.91, higher than the critical *F* value. Thus, the model was statistically significant at a 95% confidence level; it had a coefficient of determination of 90.60%. An analysis of the generated response surface (Figure 1b) in relation to the effects of the input variables temperature and acid concentration revealed that by setting the time at level +1 (60 min) there is a region that minimizes the xylose concentration, when the −1 temperature and −1 acid concentration levels are used. It is possible to infer that the concentration of xylose removed is maximum, in the studied interval, when the acid concentration and temperature are maximum.

The effectiveness of the chemical treatments was assessed based on the Kappa number, which directly reflects how much residue other than cellulose is present in the material. In other words, the lower the Kappa number, the less residue there is in the isolated cellulose. Condition 8 showed the greatest statistical significance and the greatest removal of hemicellulose. The Kappa number at the end of the process was 2.40, indicating cellulose with approximately 98% purity. Incomplete delignification of biomass materials imparts a brown coloration to the cellulosic components. During the chemical and physicochemical pretreatment methods, new chromophores are also generated from lignin and hemicellulose.^29,30^ In most cases, a bleaching treatment is necessary to increase the purity of cellulose and, consequently, to obtain more substituted CA and a higher-quality membrane at the end of the process. Because the sisal fiber had a very low Kappa number, the cellulose is closer to its pure form and thus had a whiter coloration. This is an extremely satisfactory result, as no purification step would be required before acetylation.

### 3.2 Synthesis of CA

The cellulose from sisal fiber, derived via the organosolv method, was acetylated using the homogeneous acetylation method. Based on FTIR spectroscopy, cellulose triacetate was formed, exhibiting a degree of substitution of 2.71. Literature data report that the homogeneous acetylation of cellulose derived from newsprint—involving reaction times exceeding 24 h—resulted in the formation of diacetate with a degree of substitution of 2.27.^31^ Researchers utilizing sugar cane bagasse as a cellulose source obtained cellulose triacetate with a degree of substitution of 2.52 through homogeneous acetylation.^22^ Although both studies also employed biomass as a cellulose source, their results were inferior to those obtained in the present work; furthermore, it is noteworthy that the cellulose used here which contained a minor degree of contamination (approximately 2.52%) facilitated a more effective substitution of hydroxyl groups by acetyl groups, thereby leading to the formation of cellulose triacetate.

Figure 2 shows the FTIR spectrum of CA. The peaks in 1740 and 1044 cm^–1^ are attributed to acetyl groups (C=O) and stretching vibrations of – OH and C–O–C in anhydroglucose units. The peak at 1370 cm^–1^ is due to C–H deformation, and the peak at 1230 cm^–1^ is from C–O stretching in acetyl groups.^32,33^ There is a notable reduction in transmittance intensity in the region of 3330 cm^–1^. This can be attributed to a reduction in the amount of hydroxyl groups in the cellulose chains because the acetylation reaction replaces hydroxyl groups with acetyl groups.^34^ The new peaks that appeared after acetylation are characteristic and consistent with those of pure CA.^32^

**Figure 2:**
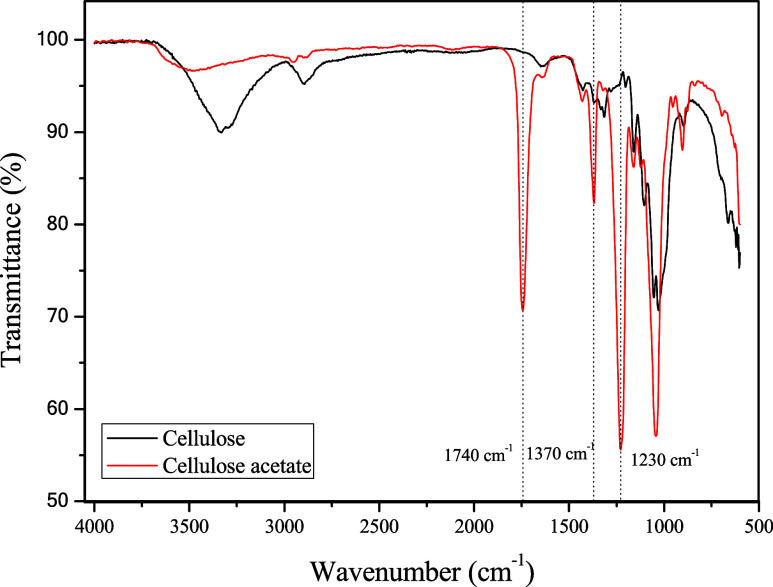
FTIR spectrum of CA and cellulose recovery from sisal fiber.

### 3.3 CA Membrane Performance

The CA membrane performance was evaluated based on porosity, permeate vapor flux, and furfural removal. Table 2 shows how the presence of PEG 400 in the membrane affected these properties.

**Table 2:** Experimental Conditions and Physical Parameters of Sisal Fiber CA Membranes

membrane	PEG 400 (w%)	synthesis temperature (°C)	Porosity (%)	water vapor flow (mg h^–1^ cm^–2^)	furfural removal (%)
M1	-	5	51.23 ± 0.31	4.24 × 10^–2^ ± 0.08	89.32 ± 0.62
M2	-	25	38.10 ± 0.27	7.77 × 10^–2^ ± 0.07	86.37 ± 0.45
M3	2	5	72.78 ± 0.41	3.24 × 10^–2^ ± 0.02	92.01 ± 0.35
M4	2	25	71.10 ± 0.45	4.36 × 10^–2^ ± 0.09	90.82 ± 0.21

Based on scanning electron microscopy and atomic force microscopy, all membranes had pores. Membrane M3 presented the highest porosity (72.78%), but it had a low permeate vapor flux (Table 2). This indicates that despite having many pores, they were smaller in size compared with other membranes, such as membrane M2, which had a high-water vapor flux (7.77 × 10^–2^ mg h^–1^ cm^–2^) but a lower porosity than membrane M3. This can also be observed in the micrograph of membrane M3, the only one for which pores could not be visualized. The inclusion of PEG 400 as an additive improves mechanical properties, increasing the hydrophilicity of the surface to control the distribution and size of the pores.^35^

Membranes M3 and M4, which included PEG 400, presented the best porosity, with an approximately 1.5% difference between them. The synthesis temperature may be the determining factor for the differences in pore size and, consequently, a lower permeate vapor flux, given that the smaller the pore size, the greater the selectivity for inhibitors such as furfural.^36^ Membrane M3, which used a coagulation bath temperature of 5 °C but included PEG 400 as an additive, could remove approximately 92% of the furfural contained in the initial hydrolyzed liquor. Membrane M1, synthesized with the same coagulation bath temperature but lacking PEG 400, could remove 89% of furfural. This value is like membrane M4, a finding that corroborates the importance of temperature in membrane production using the phase inversion method. Polymeric membranes containing PEG to enhance their selectivity exhibit macrovoid growth and, consequently, smaller pore sizes.^37^ Research using biomass to produce CA membranes has reported a similar permeate water flux. Membranes produced with newspaper pulp^38^ had a permeate water flux of 3.4 × 10^–5^ g s^–1^ cm^–2^. Membranes produced using coconut shells^29^ had a vapor permeability of 6.61 × 10^–5^ g s^–1^ cm^–2^. These values are lower than those obtained for sisal fiber membranes.

For the furfural rejection and retention tests, a lignocellulosic liquor produced from the biomass itself, containing 5.39 g L^–1^ glucose, 11.09 g L^–1^ xylose, and 5.57 g L^–1^ furfural, was used. Membrane M3 presented the highest furfural retention percentage (92%). Based on the physical properties and the rejection/retention values, the synthesis conditions with PEG 400 in the polymer structure and lower temperatures produced membranes with the best ability to remove furfural. Membranes M1 and M4 presented furfural rejection and retention percentages above the average reported in the literature, with approximately 90% of the furfural contained in the initial hydrolysate (Table 2). For comparison, studies that evaluated membranes in detoxification processes with common hydrolyzed liquor reported 60%–80% furfural retention under normal operating conditions.^36,39^ The values obtained in the present study also stand out when compared with techniques applied in industry, namely fractional distillation, which has <60% recovery.^40^ Factors such as analyte concentration in the solution to be tested, pore size, and physicochemical interactions can define the effectiveness of the separation process.

### 3.4 Morphology of the CA Membranes

A set of characterization tests was used to evaluate the effect of PEG 400 addition on the performance and physical and chemical properties of the CA membranes. The membranes lacking PEG 400 had a transparent appearance, while the others presented an opaquer appearance with an average thickness of 0.16 mm. As shown in Figure 3, the surfaces of the CA membranes presented different morphologies during the synthesis process, but all possessed an asymmetric structure. The scanning electron micrographs show the side that was in contact with the air and the side that was in contact with the coagulation bath during synthesis. In general, the textural differences among the membranes can be correlated to factors such as the bath temperature, the polymer concentration, the presence and nature of additives, and the solvent type.^41^

**Figure 3:**
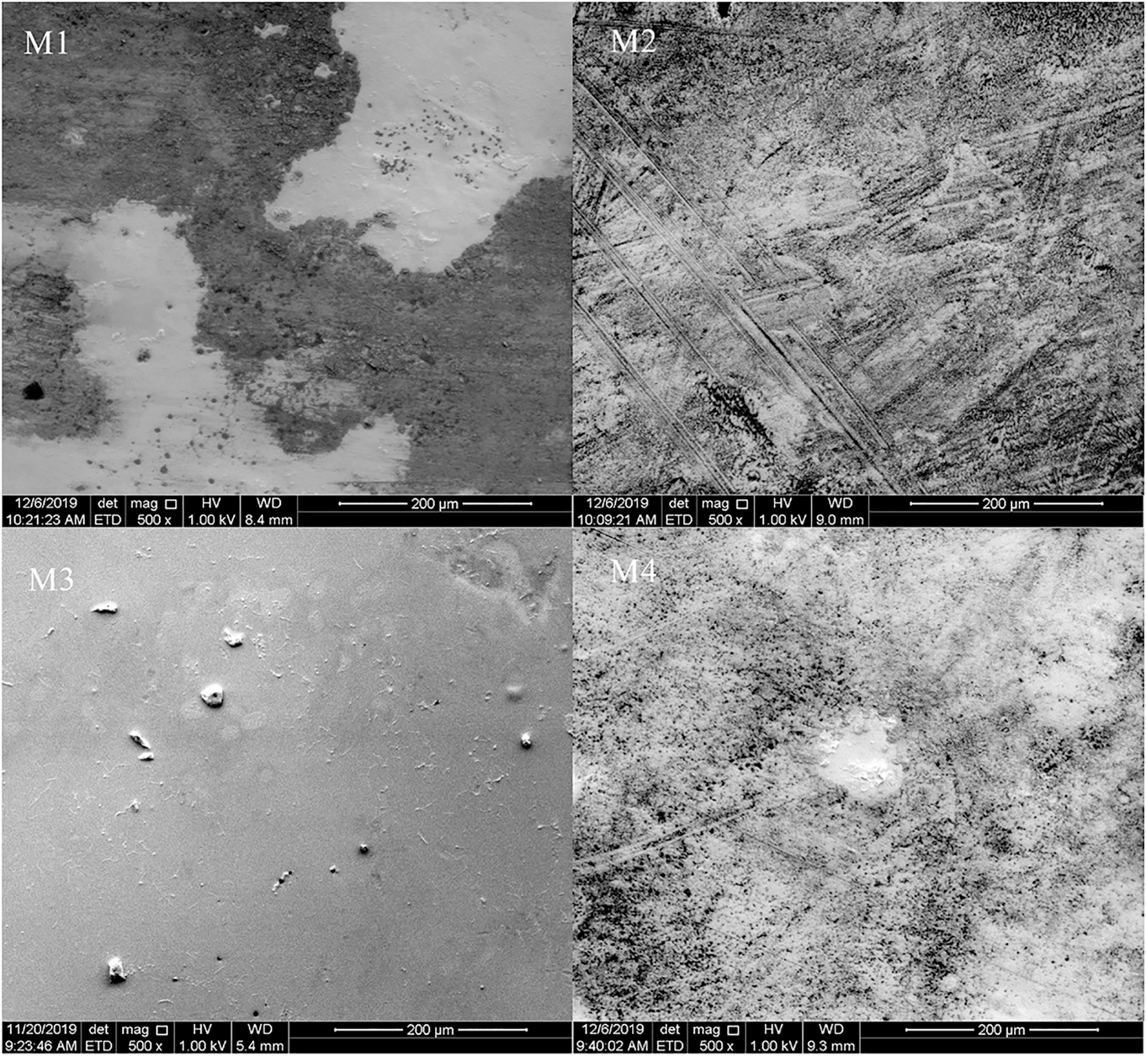
Scanning electron micrographs of the sisal fiber CA membranes. M1 and M2 are pure CA, whereas M3 and M4 contain CA and PEG 400.

The morphology of membranes M3 and M4—both containing PEG 400—suggests that the coagulation bath temperature was a determining factor in their structure. Membrane M3 presented a denser appearance, while membrane M4 presented a porous surface. PEG increases viscosity and acts as a pore-forming agent.^42^ Nevertheless, temperature likely influenced the denser surface characteristics. Most of the CA membranes exhibited a porous and asymmetrical morphology. For membranes M1 and M2, without PEG 400, the polymer matrix was not fully dissolved in the solvent, resulting in distinctly colored polymers on the surface, and areas that did not react during the phase inversion process. Membrane M4 included CA residues that did not react with the solvent on the membrane surface. Overall, some CA membranes exhibited a morphology that indicated the CA polymer matrix was not fully dissolved in the solvent, resulting in asymmetric membranes with porous and dense regions. This morphology is consistent with previous studies that suggest that the presence of lignin fragments or other interferents affected the interactions between the polymer chains, resulting in low-density regions in the polymer.^43,44^

The percentage of pores described inTable 02 illustrates their distribution across the surface. By comparing the micrographs of membranes M3 and M4 (Figure 3) with images of distinct regions of the same membranes at 1000× magnification (Figure S1), we can confirm that the membrane does not exhibit a uniform morphology, alternating between dense and porous regions—the latter featuring pores of varying sizes. In light of the permeated vapor flux parameters and other data, it is suggested that the low permeated vapor flux value observed for membrane M3 indicates that, despite possessing a high number of pores, these pores are smaller in size compared to those of the other membranes—thereby corroborating the porosity percentage data obtained.

Regarding the recovery of furfural using cellulose acetate membranes, the liquor resulting from acid hydrolysis was permeated through the analyzed membranes; among the analytes present, furfural exhibited the highest recovery rates, reaching approximately 92% for membrane M3 under the optimal conditions studied. The quantification of the recovered furfural was performed using High-Performance Liquid Chromatography (HPLC) equipped with a DAD detector at a wavelength of 280 nm for all samples (Figures S2–S3).

Figure 4 shows the atomic force micrographs of the CA membrane surfaces. The micrographs demonstrate that membrane surface roughness correlates with pore formation. Membrane pores or valleys are indicated by dark areas and high spots, and ridges by bright regions. The addition of PEG 400 to membranes M4 and M3 contributed to better polymer dissolution in the solvent, resulting in a more uniform surface with fewer pores compared with membranes M1 and M2. This finding is consistent with the scanning electron micrographs in Figure 3, which show that despite the presence of pores, the polymer matrices for membranes M1 and M2 were not fully solubilized.

**Figure 4:**
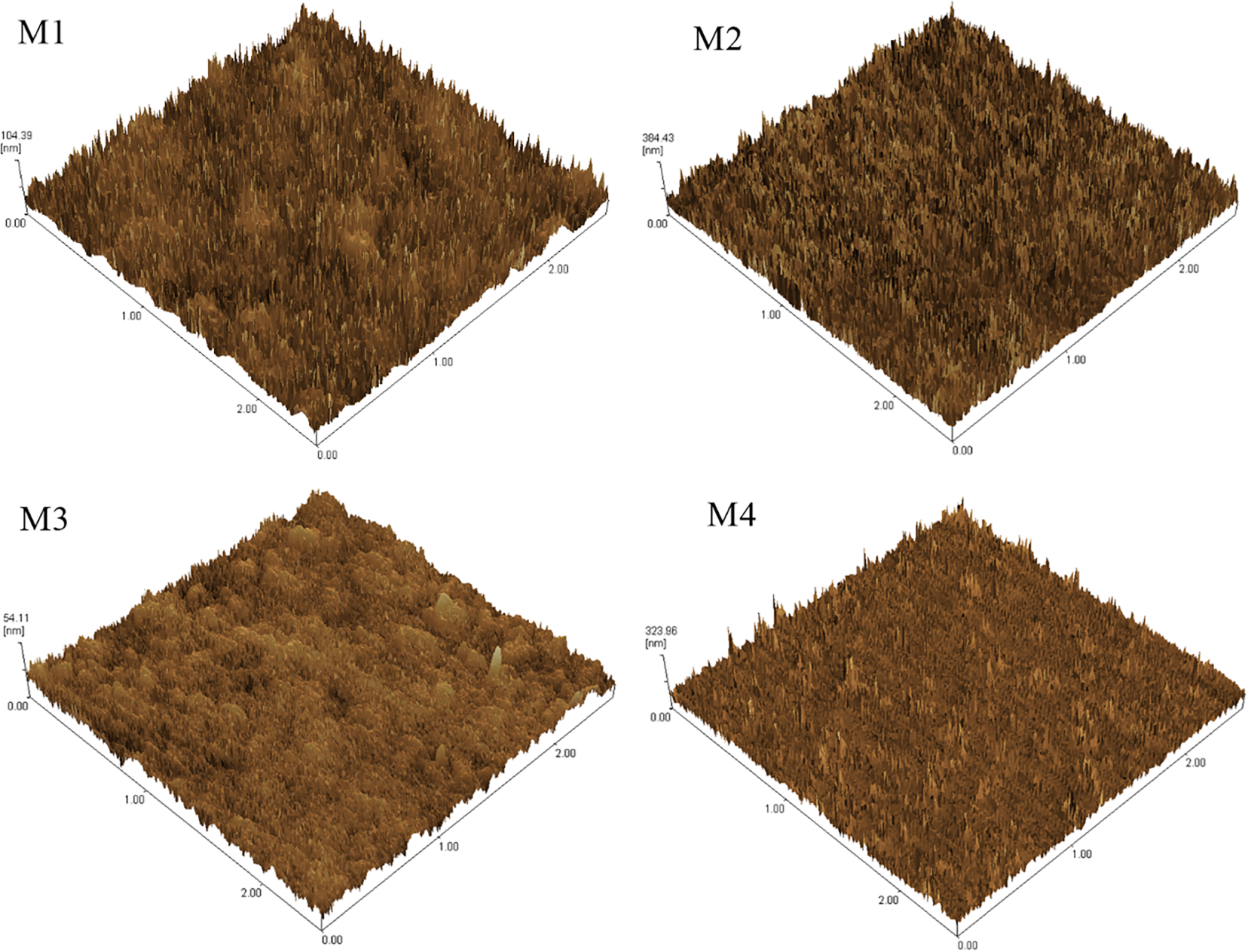
Atomic force micrographs of sisal fiber CA membranes. M1 and M2 are pure CA, whereas M3 and M4 contain CA and PEG 400.

Based on the data, the membranes containing PEG 400 had the best properties. In most cases, the addition of more PEG will increase the proportion of the polymer-poor phase, resulting in the formation of more pores during the glass transition phase and, consequently, greater solvent flux. This is one of the key steps during the phase inversion process, where physicochemical processes produce the porous structure of the membrane. At the maximum PEG dose, a polymer-poor phase is intertwined with a continuous polymer-rich phase, resulting in denser membranes; smaller, narrower, and more distributed, interconnected pores are formed in the membrane matrix.^41^ This explains the process that occurred in membranes M3 and M4: They have fewer pores mainly due to the greater amount of PEG 400 in their structure, resulting in a dense morphology in some regions and smaller pores. The data from the other tests corroborate the morphological findings.

## 4 Conclusions

The cellulose isolation step plays a fundamental role in the quality of the membrane produced, as the presence of any contaminants in the CA impairs its dissolution in the solvent. Consequently, the membranes presented porous zones alternating with denser regions, an observed in membranes M1 and M2. Under the best conditions, the recovered cellulose contained only 2.4% residues. Acid pretreatment led to a xylose removal concentration of 14.8 g L^–1^, highlighting the benefits of sisal fiber in terms of its carbohydrate content and the effectiveness of hemicellulose removal. When acetylated, cellulose from sisal fiber formed cellulose triacetate, as confirmed with spectroscopic analysis.

Pores distributed throughout the surface of the CA membranes, with >70% porosity for membranes M3 and M4. The inclusion of PEG 400 combined with a lower coagulation bath temperatures during synthesis were the most influential factors in achieving satisfactory retention of furfural. Membranes M3 and M4, both containing PEG 400, presented the best performance: Membrane M3 retained 92% of furfural and membrane M2 had the highest permeate flux at 7.77 × 10^–2^ mg h^–1^ cm^–2^. Overall, this study has demonstrated the utility of sisal fiber to produce cellulose triacetate and CA membranes for furfural recovery.

## Supplementary Material



## Notes

Scanning electron micrographs of the sisal fiber membranes: M3 and M4 contain CA and PEG 400 - distinct regions of the same membranes at 1000× magnification (Figure S1); chromatograms of the reference lignocellulosic hydrolysate liquor (LP) and the liquors that permeated the membranes (Figure S2); chromatograms of the reference lignocellulosic hydrolysate liquor and the liquors that permeated the membranes (Figure S3) (PDF)
