# Supplementary material for: Cellulose Acetate Membranes from Sisal Fiber Applied for Furfural Recovery
Source: ACS Omega. 2026 Apr 29;11(18):27159–67. doi: 10.1021/acsomega.6c00887 (PMC13176985; doi:10.1021/acsomega.6c00887)
Supplement: Supplementary file 1 [file ao6c00887_si_001.pdf]

Cellulose Acetate Membranes from Sisal Fiber applied for Furfural Recovery

Franklin Damião Xavier[1], Maria Gardennia Fonseca[2], Bruno Alessandro Silva Guedes Lima[3], Sandro Marden Torres[3], Marta Maria Conceição[*, 4]

[1] PPGQ/CCEN, Universidade Federal da Paraíba, João Pessoa-PB, Brazil;

[2] Departamento de Química/CCEN, Universidade Federal da Paraíba, João Pessoa-PB, Brazil;

[3] Departamento de Engenharia Mecânica/CT, Universidade Federal da Paraíba, João Pessoa-PB, Brazil;

[4] Departamento de Tecnologia de Alimentos/CTDR, Universidade Federal da Paraíba, Av. dos Escoteiros, sn. Mangabeira VII, 58058-600 João Pessoa-PB, Brazil

*Corresponding author: martamaria8@yahoo.com

Supplementary information

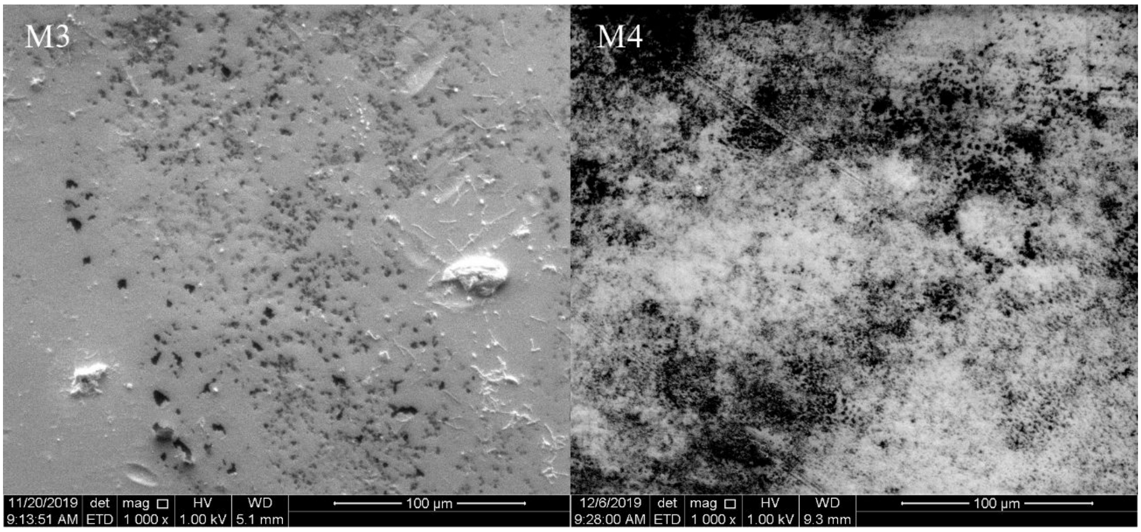

Figure S1 Scanning electron micrographs of the sisal fiber membranes: M3 and M4 contain CA and PEG 400 (distinct regions of the same membranes at 1000x magnification)

The percentage of pores described in Table 02 illustrates their distribution across the surface. By comparing the micrographs of membranes M3 and M4 (Figure 3) with images of distinct regions of the same membranes at 1000x magnification (Figure S1), we can confirm that the membrane does not exhibit a uniform morphology, alternating between dense and porous regions—the latter featuring pores of varying sizes. In light of the permeated vapor flux parameters and other data, it is suggested that the low permeated vapor flux value observed for membrane M3 indicates that, despite possessing a high

number of pores, these pores are smaller in size compared to those of the other membranes—thereby corroborating the porosity percentage data obtained.

Regarding the recovery of furfural using cellulose acetate membranes, the liquor resulting from acid hydrolysis was permeated through the analyzed membranes; among the analytes present, furfural exhibited the highest recovery rates, reaching approximately 92% for membrane M3 under the optimal conditions studied. The quantification of the recovered furfural was performed using High-Performance Liquid Chromatography (HPLC) equipped with a DAD detector at a wavelength of 280 nm for all samples (Figure S2). Presented below are the chromatograms comparing the reference lignocellulosic hydrolysate liquor (LP) with the liquors that permeated the membranes following each trial.

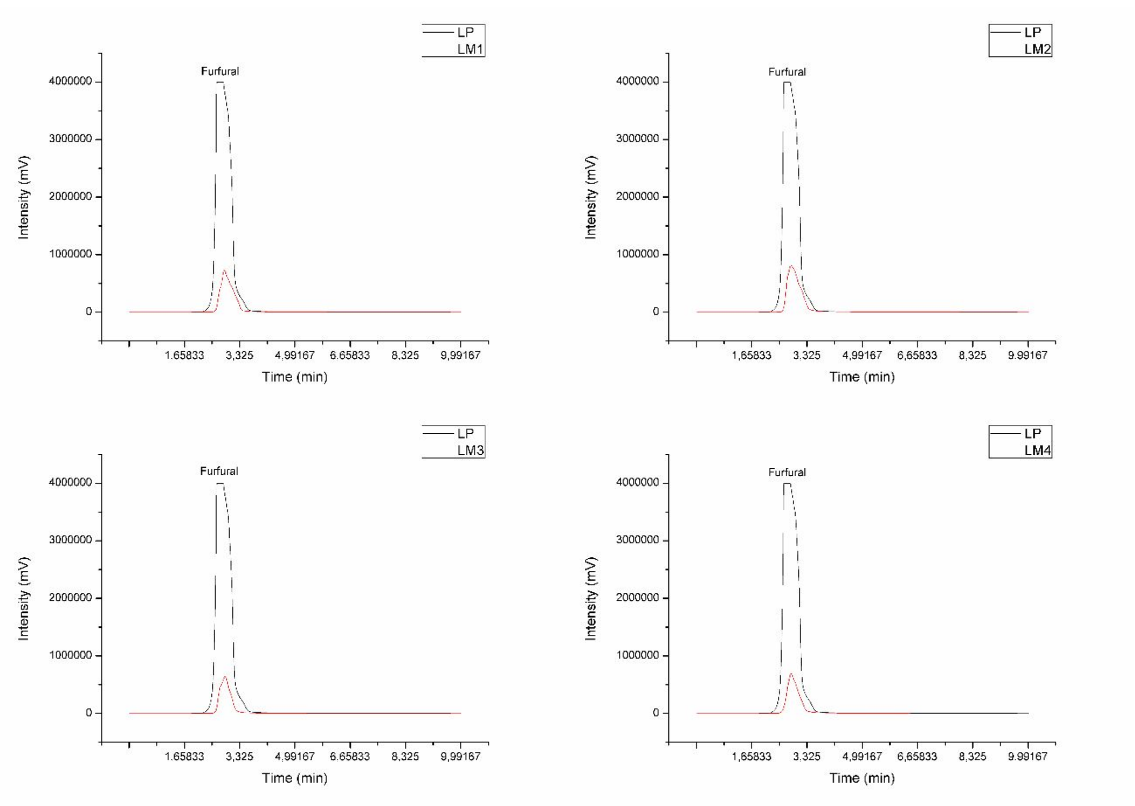

Figure S2 Chromatograms of the reference lignocellulosic hydrolysate liquor (LP) and the liquors that permeated the membranes

The LP liquor is the lignocellulosic hydrolysate of sisal obtained after the acid hydrolysis of the biomass, which was used as a reference for all membrane retention tests. In the chromatograms, the region where furfural was plotted was highlighted, showing a retention time of 2.9 minutes in accordance with the analytical standard used in the respective method. The highlighted chromatograms corroborated the data presented in Table 02, wherein the liquor permeated through membrane M3 exhibited the lowest furfural concentration—signifying, therefore, a higher recovery in the non-permeated

fraction (Figure S3). Presented below is the comparative chromatogram of all permeated liquors relative to the reference liquor.

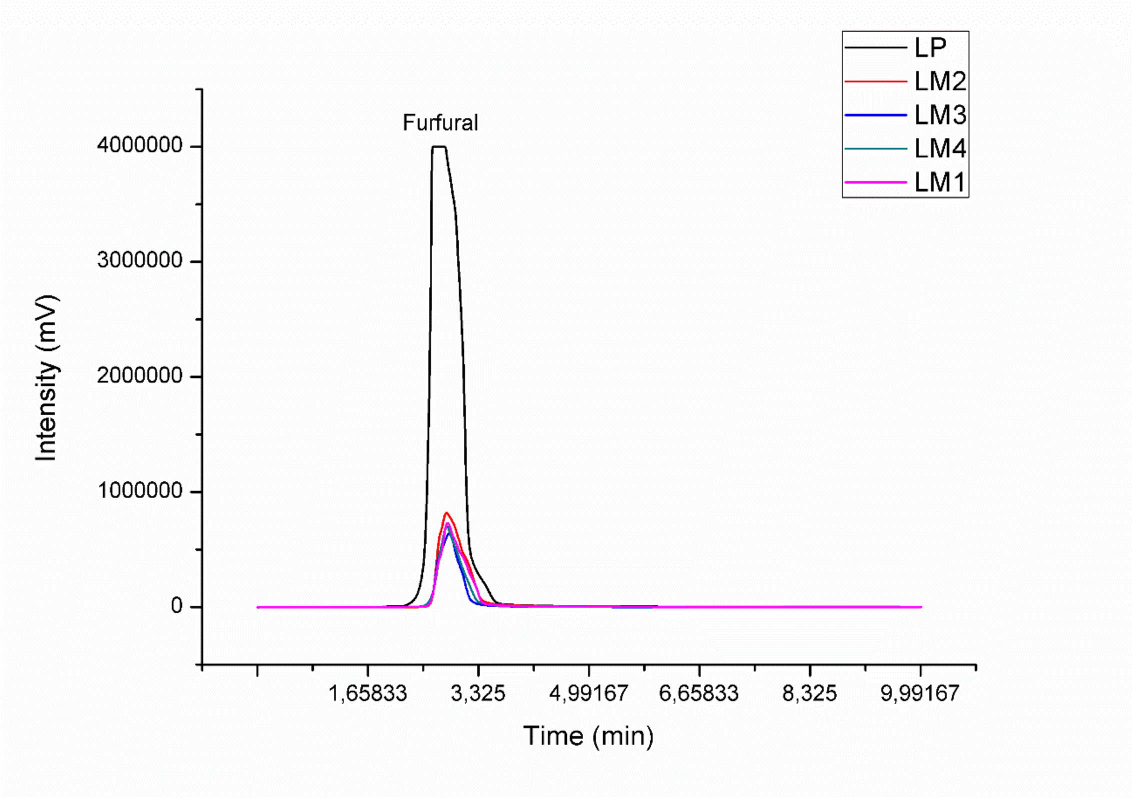

Figure S3 Chromatograms of the reference lignocellulosic hydrolysate liquor and the liquors that permeated the membranes
